# Quantitative Secondary Ion Mass Spectrometry

**DOI:** 10.6028/jres.093.140

**Published:** 1988-06-01

**Authors:** M. Grasserbauer

**Affiliations:** Institute for Analytical Chemistry, Laboratory for Physical Analysis, Technical University Vienna, Getreidemarkt 9, A-1060 Wien, Austria

## 1. Introduction

Secondary Ion Mass Spectrometry [SIMS] provides a unique analytical potential due to the fact that all elements can be analyzed with high sensitivity and isotopic specificity, that elemental and molecular (bonding) information can be gained and that micro, surface and bulk analysis can be performed. The major limitations of SIMS are determined by the complexity of the mass spectra, the large variation of the secondary ion yields for different elements, and also for a particular element in different matrices (chemical matrix effect), and by the disorder induced into the analytical zone due to the high energy (*E*_0_=1−20 keV) of the primary ions [1,2].

To overcome these limitations *in praxi*, the following approach for quantitative trace analysis is most useful:

- Use of reactive ion sputtering (oxygen or cesium) to increase the secondary ion yield and to achieve a chemical modification of the surface zone which reduces the chemical matrix effects.
- Use of high performance instrumentation which allows the identification (and separation) of interfering species by high mass resolution.
- Quantitation with relative sensitivity factors (RSFs) obtained from external or internal calibration.
- Systematic study of all sources of analytical errors, particularly of the measurement process, as described by Powell [3].
- Development of problem oriented measurement techniques and analytical strategies, combining SIMS with other analytical techniques yielding confirmatory and supplementary information (example ref. [4]).

Quantitation procedures and the general problems associated with quantitation of secondary ion mass spectra are discussed in several excellent publications [1,2,5–7]; thus this paper will concentrate on specific tasks of quantitative analysis with SIMS. These tasks are: multielement, ultratrace analysis of metals; high-accuracy, depth distribution analysis of dopants in silicon; and quantitative interface analysis.

## 2. Multielement Ultratrace Analysis of Metals

The need arises mainly from microelectronics, with its extreme purity requirements for metals used for the production of electrode lines and interconnects in VLSI devices: In refractory metals used for 1 Mbit and 4 Mbit storage devices, alkali elements and U, Th must not exceed 10 ng/g; the concentration of transition metals must be below 100 ng/g.

Due to the limited potential of NAA for ultratrace analysis of refractory metals, only mass spectrometric techniques can be considered for direct solid state analysis. A comparison of the useful yields [8] (SSMS : 10^−8^−10^−7^, GDMS 10^−7^, SNMS 10^−9^, SIMS 10^−1^−10^−5^ [using reactive ion bombardment]) shows the large basic potential of SIMS.

**Figure 1:**
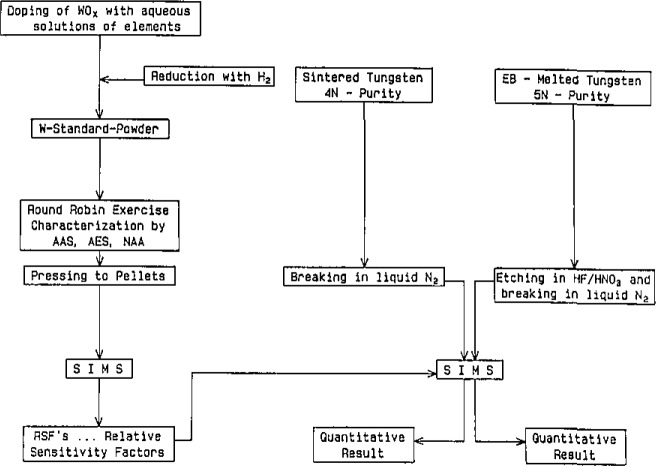
Scheme for quantitative multielement ultratrace analysis of refractory metals (example: tungsten) with SIMS [9].

The need to establish RSFs with reference materials characterized for trace elements—a disadvantage compared to SSMS, GDMS and SNMS—makes the analytical procedure more elaborate. Figure 1 contains the basic steps for the preparation and characterization of the reference material (doping with 40 elements, in the *μ*g/g-range, round robin analysis, test of homogeneity with SIMS) and the analysis of the materials [9].

Thirty elements are analyzed with oxygen primary ions, 10 with cesium. Interferences are eliminated by energy filtering, peak stripping and high mass resolution. Several mass spectra or depth profiles are recorded to obtain a fairly representative value within a reasonable time (ca. 2 hours). Material consumption per analysis is ca. 1 *μ*g (1–10 mg for SSMS, 1–10 mg for GDMS). With optimized measurement techniques, detection limits (calculated from the analysis of the reference materials) between 1 pg/g and 100 ng/g are obtained with SIMS (SSMS 10–100 ng/g, GDMS 1–10 ng/g) [9–11].

For the assessment of accuracy, chemical matrix effects were studied by comparing results obtained with oxygen bombardment (with and without saturation) and cesium bombardment and using different external standards (doped materials and ion implants). The values lie typically within a factor of 2 [10]. Furthermore, a comparison of SIMS with GDMS and SSMS (including NAA results on a few elements) for W and Mo showed that the agreement of SIMS with the other methods is on the same order (typically within a factor 2) as between GDMS, SSMS and NAA, respectively. This indicates the absence of large systematic errors in the SIMS results due to a chemical matrix effect. It was found that the major source for deviations between the different methods is the inhomogeneity of the materials analyzed. This again shows the need for reference materials that are homogeneous on the nm to cm scale.

For homogeneous materials, an analytical accuracy of SIMS for the ultratrace analysis of U and Th in aluminum of 10% has been reported [12]. This corresponds well with the experiences obtained in the analysis of semiconductors.

**Figure 2:**
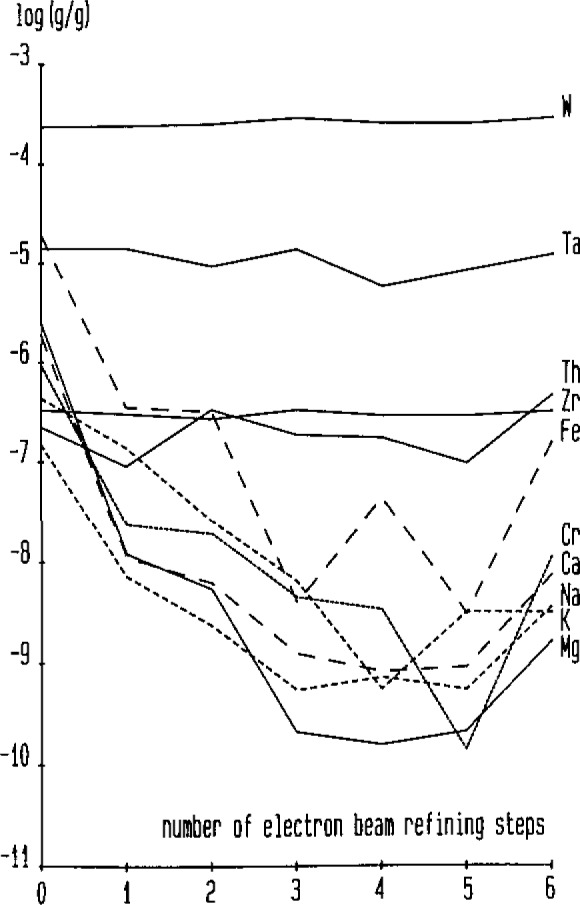
Quantitative ultratrace analysis of impurities in molybdenum with SIMS: Relation between impurity concentration and numbers of electron beam refining steps [11].

With these figures of merit, SIMS can be considered as another major technique for ultratrace analysis. It is—up to now—the only method for ultratrace analysis in thin films—a task which is of great interest to study the process related contamination of metallization layers. As an example for the application of SIMS, figure 2 shows the ultratrace analysis of the impurity content in molybdenum as a function of the number of electron beam refining steps. The increase of impurity levels after the 6th step is due to the inhomogeneity on the cm-scale of the cast molybdenum [11].

## 3. High-Accuracy, Depth Distribution Analysis of Dopant Elements in Silicon

SIMS is presently the most important technique for distribution analysis of dopant elements in semiconductors [4,13–17]. For silicon under optimized conditions (table 1), detection limits in the range between 10^14^ and 3×10^15^ cm^−3^ can be obtained. The accuracy of the concentration values in a profile is on the order of 5% (B) to 20% (P); that of the depth scale ~5% (using actual crater depth measurements) (see also [13]). The accuracy of SIMS profiles can be determined in two different ways:

- Comparison of profiles quantified with the internal calibration (“fluence”) method with those obtained using external (homogeneous) reference materials. Fluence measurements by an independent technique like NAA or RBS are valuable to check the accuracy of fluence values obtained from measurement of the implantation current.
- Comparison of profiles obtained with different techniques exhibiting independent systematic errors [16,19]. Such comparative analyses are of great significance to study the artefacts [18] which occur in SIMS depth profiling. For all dopant elements, only electrical measurements [e.g., spreading resistance (SR)] can be applied to annealed specimens. There is however a systematic deviation between elemental and electrical profiles at concentrations above the (electrical) solubility limit and at the deeper side of the profile, for which SR profiles seem to be steeper. Comparison of elemental profiles is even more difficult: only for ^10^B a “reference technique”—NAA exists which exhibits the sufficient accuracy—at least for annealed specimens [19]. For non-annealed materials, differences between SIMS and NAA depth profiles for B in silicon have been observed [20]. For the dopant elements As, P, and Sb, chemical etching has to be applied for NAA depth profiling. Moderate depth resolution and rather large analytical errors are encountered. For Sb, RBS can be applied for depth profiling. Although the method is considered to be largely free from systematic errors, it exhibits only a rather poor depth resolution and a high detection limit. Thus the depth profiles obtained with RBS can only be compared with SIMS profiles in a very limited manner.

Summarizing this discussion of the assessment of the accuracy of SIMS depth profiles (which is presented in detail in ref. [16]), it must be stated that there is a significant lack of independent methods to study SIMS artefacts. The most successful approach still seems to be the systematic investigation of ion-solid interactions, thus creating a broader understanding for physical processes like sputtering and secondary ion formation [21].

Up to this point the discussion has been concentrating on “pure” silicon matrices. In industrial practice the combined system SiO_2_/Si is usually of great interest. This means depth profiling through an insulator/semiconductor system with a changing matrix. Compensation of charging and matrix effects is necessary to achieve quantitative results. This problem is especially serious if an element must be measured at high mass resolution, like P (*M*/Δ*M ~* 4500). New techniques are necessary to obtain stable and accurate profiles through the layer system SiO_2_/Si [22]: They apply computerized peak centering routines in switching between references mass and analytical ion to eliminate magnetic drift and hysteresis effects. Secondly, a complete charge compensation is achieved by measurement of the intensity of the reference ion (^30^Si^+^) at the steep flank of the energy of distribution. This intensity value is extremely sensitive to charging: a surface charge of only 1 volt influences the reference intensity by 10%. Changes in this reference ion intensity are used to monitor the process of charge compensation by adjustment of the sample potential. The third step—compensation of the chemical matrix effect—is performed by oxygen saturation of the sample surface during analysis. To control the degree of surface saturation the secondary ion ratio SiO_2_^+^/Si_2_^+^, which is extremely sensitive towards oxygen coverage of the surface, is measured. With this procedure it is possible to control the compensation of the chemical matrix effect eight times more accurately than by monitoring only atomic ions (e.g., the reference mass).

**Figure 3:**
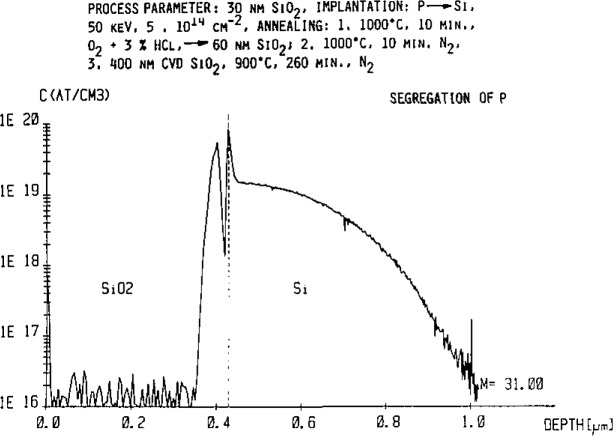
Quantitative phosphorus profile measured at high mass resolution (*M*/Δ*M* = 4500) in the SiO_2_/Si system for determination of the segregation coefficient.

These extensive computer based measurement techniques finally allow the measurement of the distribution of P across thick oxide films into the silicon matrix with high accuracy (estimated to be~30% for a point on the profile) for determining the technically very important assessment of the segregation of this dopant element during oxidation of silicon in device manufacturing (fig. 3).

Distribution analysis of dopant elements in semiconductors by SIMS is by far not fully developed yet. Due to the increasing demands posed by semiconductor technology new challenges arise, e.g., for simultaneous multielement depth profiling, for which new computerized measurement approaches have to be established [23], or in high lateral resolution dopant analysis in devices with finely focused ion beams [24], or for high depth resolution profiles of very shallow or multilayer structures [25], or finally the problem of quantitation through layers of changing matrices [26].

## 4. Quantitative Interface Analysis

**Figure 4:**
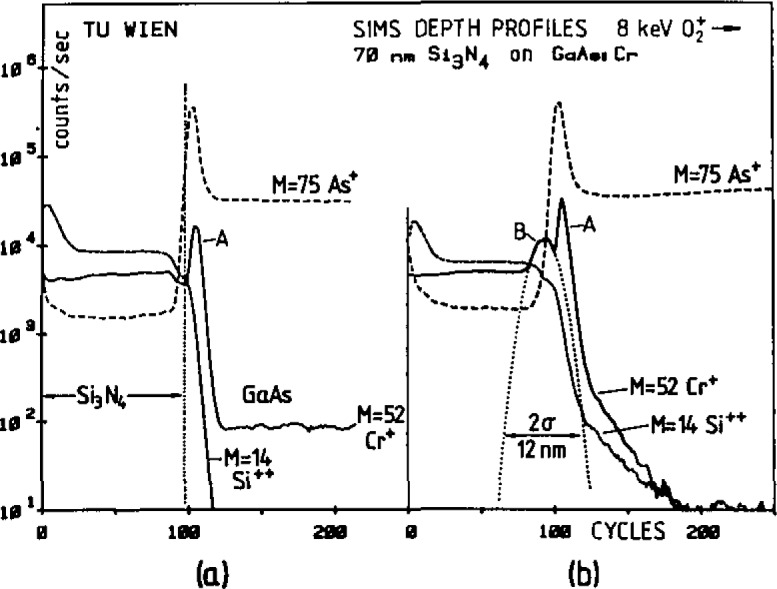
Depth profiles of 70 nm Si_3_N_4_/GaAs doped with 3.5×10^16^cm^−3^ Cr. Left: before annealing. Right: after annealing. Primary ions: oxygen [28].

Quantitative analysis at interfaces is subject to pronounced artefacts and erroneous results due to cascade and recoil mixing, changes in sputter rates and ionization yields and ion beam induced diffusion (e.g., for alkali elements) [27]. Extensive investigations are usually necessary to achieve a quantitative result, as shown here in an exemplary manner for the study of the distribution of the dopant element Cr between GaAs and the covering Si_3_N_4_ layer [28]. Figure 4 shows the SIMS profiles of ^52^Cr^+^, ^28^Si^2+^ and ^75^As^+^ obtained with oxygen primary ions in a GaAs material before and after annealing. Cr peaks are observed in GaAs adjacent to the interface (A) in both cases and in the Si_3_N_4_ layer (B) after annealing. Ion implantation of Cr into a Si_3_N_4_/GaAs structure also showed these two peaks, indicating that peak A was an artefact due to chemical yield enhancement by oxygen at the interface analysis of the annealed sample. Analysis with Ar^+^ primary ions showed only peak B, thus raising the question about the origin of chemical signal enhancement. Modelling of the primary ion implantation and sputter process yielded the origin of peak B. Due to the higher stopping power of GaAs compared to Si_3_N_4_ for the primary oxygen ions, these are enriched in the near interface layer of GaAs when sputtering through the interface (enrichment factor ~2). Secondly the sputter rate of GaAs is a factor of 3.3 higher than that of Si_3_N_4_. Both effects combined lead to a signal increase for Cr in the near-interface zone of GaAs of about a factor of 10.

Once peak A was identified as an artefact, the real distribution of Cr could be established by fitting a Gaussian profile to peak B. The accuracy of this method was tested by comparison of the amount of outdiffused Cr from GaAs with that corresponding to peak B. An agreement for both Cr values of 30% was found.

## 5. Further Challenges

**Figure 5:**
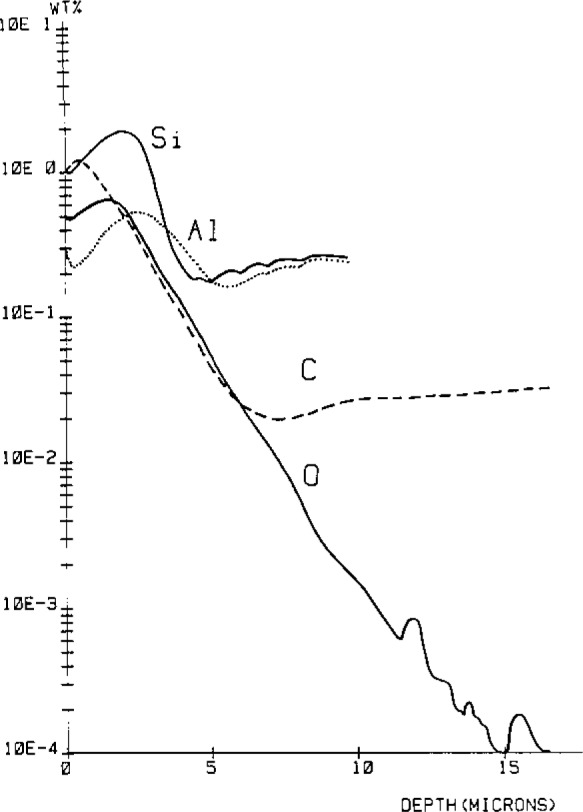
Quantitative depth profiles of C, O, Al and Si in FeAl (0.24), Si(0.25) alloy after annealing in CO-atmosphere [30].

Quantitative SIMS has by far not reached its limits. Due to the increasing necessity for quantitative distribution analysis of trace elements particularly in high technology materials [29] a strong motivation for the development of instrumentation and analytical procedures is provided. Some of the most significant areas of current and future activities are compiled in the following (subjective) selection:

- Analysis of laterally heterogeneous systems, e.g., metals with precipitates: reduction of selective sputter effects by chemical surface reactions to obtain depth profiles with a high dynamic range (fig. 5) [30], combination of ion microscopy and mass spectrometry to establish calibration curves [31].
- “Monolayer” analysis—quantitative analysis is limited by the low number of atoms available for a data point, e.g., the detection limit for Al in a thin SiO_2_ layer on silicon is ca. 1 *μ*g/g for a consumption of 0.1 atomic layer (*d*_A_=250 *μ*m)—corresponding to a removal of 10,000 Al atoms [32]. Laser resonance ionization SIMS employing high transmission TOFs promises to extend detection limits to the ng/g range or some tens of atoms, respectively [33].
- Three-dimensional quantitative distribution analysis, employing elaborate image processing techniques. The resistive anode encoder based systems [34] seem to be particularly promising. Availability of the full 3-D information will allow correlation of all information which can be gained with SIMS—a tremendous advantage for (usually complex) technical materials.
- Phase identification by quantitative evaluation of cluster ion intensities in the secondary ion mass spectrum. Pattern recognition techniques have been shown to have great value for selection of the appropriate features most typical for a particular compound or phase [35]. Identification of phases (precipitated in a matrix or at interfaces) is in principle possible even if their size is below the spatial resolution of SIMS.
- Application of chemometric techniques to extract and verify information obtained from SIMS spectra and profiles, e.g., factor analysis [36] or fuzzy theory [37] to evaluate depth profiles.
- Compilation of sputter coefficients, ionization probabilities, useful yields [38], and relative sensitivity factors. These data would be of great value for choosing optimal measurement parameters and could enable assignment of at least semiquantitative values to secondary ion intensities for systems where a suitably matched reference material is not available. Recent investigations on semiconductor [39] and refractory metal [10] matrices indicate that RFSs can be transferred from one matrix to another by the use of scaling factors.
- Development of (further) models for sputtering and ionization which reflect the physical processes occurring under reactive ion beam bombardment.

## Figures and Tables

**Table 1:** Optimized analytical conditions and practical detection limits for surface distribution analysis of dopant elements in silicon with SIMS [4]. ${\left(\frac{M}{\Delta M}\right)}_{\mathrm{pr}.}=\text{practical mass resolution used during measurement}$.

Isotope	Primary ion	Detected ion	Interfering ion	${\left(\frac{M}{\Delta M}\right)}_{\mathrm{pr}.}$	Detection limit (atoms cm^−3^)
^10^B	$O_{2}^{+}$	^10^B^+^	^30^Si^3+^	500	10^14^
^11^B	$O_{2}^{+}$	^10^B	^10^BH^+^	400	10^14^
^31^P	Cs^+^	^31^P^−^	^30^SiH^−^	4500	1·10^15^
^75^As	Cs^+^	^75^As^−^	^29^Si^30^Si^16^O^−^	4500	3·10^15^
^121^Sb	Cs^+^	^121^Sb^28^Si^−^	$C_{x}H_{y}^{-},$	400	3·10^15^
^123^Sb	Cs^+^	^123^Sb^28^Si^−^	${\mathrm{Si}}_{x}O_{y}^{-}(?)$	400	3·10^14^
